# Cerebrospinal fluid CXLC13 indicates disease course in neuroinfection: an observational study

**DOI:** 10.1186/s12974-019-1405-8

**Published:** 2019-01-19

**Authors:** Georg Pilz, Peter Wipfler, Ferdinand Otto, Wolfgang Hitzl, Shahrzad Afazel, Elisabeth Haschke-Becher, Eugen Trinka, Andrea Harrer

**Affiliations:** 1 Department of Neurology, Christian-Doppler-Klinik, Paracelsus Medical University, Salzburg, Austria; 2 Research Office, Biostatistics, Paracelsus Medical University, Salzburg, Austria; 3 Department of Ophthalmology and Optometry, Paracelsus Medical University, Salzburg, Austria; 4 Department of Laboratory Medicine, Landeskrankenhaus, Paracelsus Medical University, Salzburg, Austria

**Keywords:** CXCL13, Neuroinfection, CSF, Neuroinflammation, Disease course

## Abstract

**Background:**

The chemokine CXCL13 is an intensively investigated biomarker in Lyme neuroborreliosis (LNB). Its role in other neuroinfections is increasingly recognized but less clear.

**Objective:**

To determine the significance of CXCL13 in established central nervous system (CNS) infections other than LNB by matching cerebrospinal fluid (CSF) CXCL13 elevations with severity of the disease course.

**Methods:**

We investigated 26 patients with bacterial (*n* = 10) and viral (*n* = 16; tick-borne encephalitis, *n* = 6; varicella zoster infection, *n* = 10) neuroinfections of whom CSF CXCL13 levels were available twice, from lumbar punctures (LP) performed at admission and follow-up. As outcome classification, we dichotomized disease courses into “uncomplicated” (meningitis, monoradiculitis) and “complicated” (signs of CNS parenchymal involvement such as encephalitis, myelitis, abscesses, or vasculitis). CXCL13 elevations above 250 pg/ml were classified as highly elevated.

**Results:**

Eight of 26 patients (31%) with both bacterial (*n* = 4) and viral (*n* = 4) neuroinfections had a complicated disease course. All of them but only 3/18 patients (17%) with an uncomplicated disease course had CSF CXCL13 elevations > 250 pg/ml at the follow-up LP (*p* < 0.001). At admission, 4/8 patients (50%) with a complicated disease course and 3/18 patients (17%) with an uncomplicated disease course showed CXCL13 elevations > 250 pg/ml. All four patients with a complicated disease course but only one with an uncomplicated disease course had sustained CXCL13 elevations at follow-up. Patient groups did not differ with regard to age, time since symptom onset, LP intervals, type of infections, and anti-pathogen treatments.

**Conclusion:**

Our study revealed pronounced CXCL13 elevations in CSF of patients with severe disease courses of bacterial and viral neuroinfections. This observation indicates a role of CXCL13 in the CNS immune defense and points at an additional diagnostic value as biomarker for unresolved immune processes leading to or associated with complications.

## Introduction

The chemokine CXCL13 in cerebrospinal fluid (CSF) is considered supportive in the differential diagnosis of Lyme neuroborreliosis (LNB) [1–5]. Elevated CXCL13 in CSF, however, also occur in other inflammatory central nervous system (CNS) diseases such as bacterial and viral meningitis, encephalitis, myelitis, and CNS lymphoma [6–8] and in patients with autoimmune CNS diseases such as multiple sclerosis (MS) and autoimmune encephalitis (AIE) [9, 10]. Beside differences for amount of CXCL13 elevations, the true function and significance of this chemokine in neuroinflammation is not fully established. As potent chemoattractant of B cells to peripheral lymph follicles, CXCL13 might play a similar role in the CNS immune defense and orchestrate B cell recruitment from peripheral blood into the CSF.

In this observational study, we investigated CXCL13 in the CSF of patients with established CNS infections other than LNB and related CXCL13 concentrations in CSF at admission and for control to the clinical disease course. Our aim was to clarify and broaden our understanding of the relevance of this key chemokine in neuroinfections.

## Material and methods

This retrospective study included 26 patients collected from chart records at the Department of Neurology of the Paracelsus Medical University during the years 2015–2017, and was approved by the local ethics committee of Salzburg (415-E/2218/5-2017). Inclusion criteria were discharge diagnosis of bacterial or viral neuroinfectious disease and CXCL13 determined twice, from CSF collected at admission and one follow-up lumbar puncture (LP). The neuroinfectious diseases were bacterial meningoencephalitis (*n* = 10; meningitis, *n* = 6; meningitis and CNS abscess, *n* = 3; meningitis and CNS vasculitis, *n* = 1), tick-borne meningoencephalitis (TBE, *n* = 6; meningitis, *n* = 4; encephalitis, *n* = 2), and varicella zoster virus (VZV) infections (*n* = 10; meningitis, *n* = 6; monoradiculitis, *n* = 2; monoradiculitis and CNS vasculitis, *n* = 1; encephalomyelitis, *n* = 1). All patients with bacterial infections received antibiotics; eight of ten patients with VZV infections received aciclovir.

**Fig. 1:**
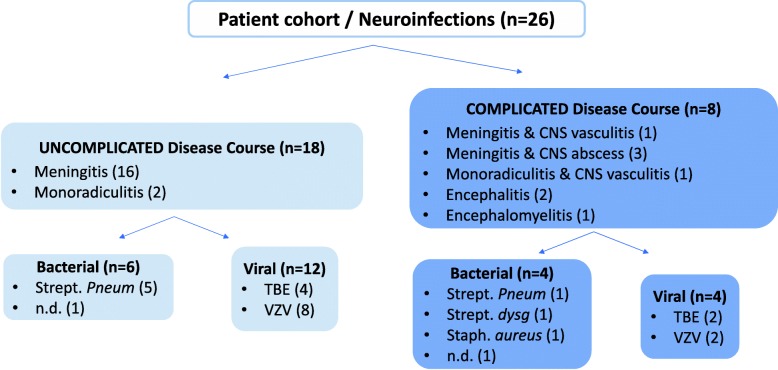
Overview of patient stratification according to disease course. *Abbreviations*: dysg, dysgalacticae; n.d., not defined; pneum., pneumoniae; Staph., *Staphylococcus*; Strept., *Streptococcus*; TBE, tick-borne encephalitis; VZV, varicella zoster virus infection

We dichotomized patients according to their disease course into groups “uncomplicated” (meningitis, monoradiculitis) or “complicated” (signs of CNS involvement such as encephalitis, myelitis, abscess, or vasculitis). Figure 1 provides a detailed overview of the patient stratification including information on the type of infection and associated causative pathogens.

CSF laboratory diagnostics including CSF cell count, cell differential (predominance of granulocytes or lympho- and monocytes), CSF/serum albumin quotient (QAlb), pathogen detection, and determination of CXCL13 (Euroimmune) were performed at the Department of Laboratory Medicine of the PMU, Salzburg. We applied the 250-pg/ml cut-off for defining highly elevated CSF CXCL13, as this is our local cut-off for supporting the diagnosis of LNB established by ROC analysis of regional patients (unpublished data) and in line with literature [2, 11].

Numerical variables (patient characteristics) were summarized with medians and ranges, and categorical variables with frequencies and percentages. Non-parametric Mann-Whitney *U* exact test (numerical variables) and chi square test (categorical variables) were applied for comparisons between groups. Receiver operating characteristics (ROC) analysis was used to estimate the performance of CXCL13 in discriminating between disease courses (complicated/uncomplicated), and the area under the curve (AUC), 95% confidence intervals (CI), *p* value (AUC 0.5), sensitivity, and specificity were computed. A *p* value < 0.05 was considered as statistically significant. Computations were done using SPSS Statistics 24.0 (IBM Germany GmbH) and Microsoft Excel (Microsoft Office2007, Redmond, USA).

## Results

Eighteen of the 26 patients (69%) had an uncomplicated and eight patients (31%) had a complicated disease course (Fig. 1). There was no significant difference in gender, age, type of infection (bacterial/viral), time since symptom onset, interval between the two LPs, and anti-pathogen treatment between groups (Table 1).

**Table 1:**
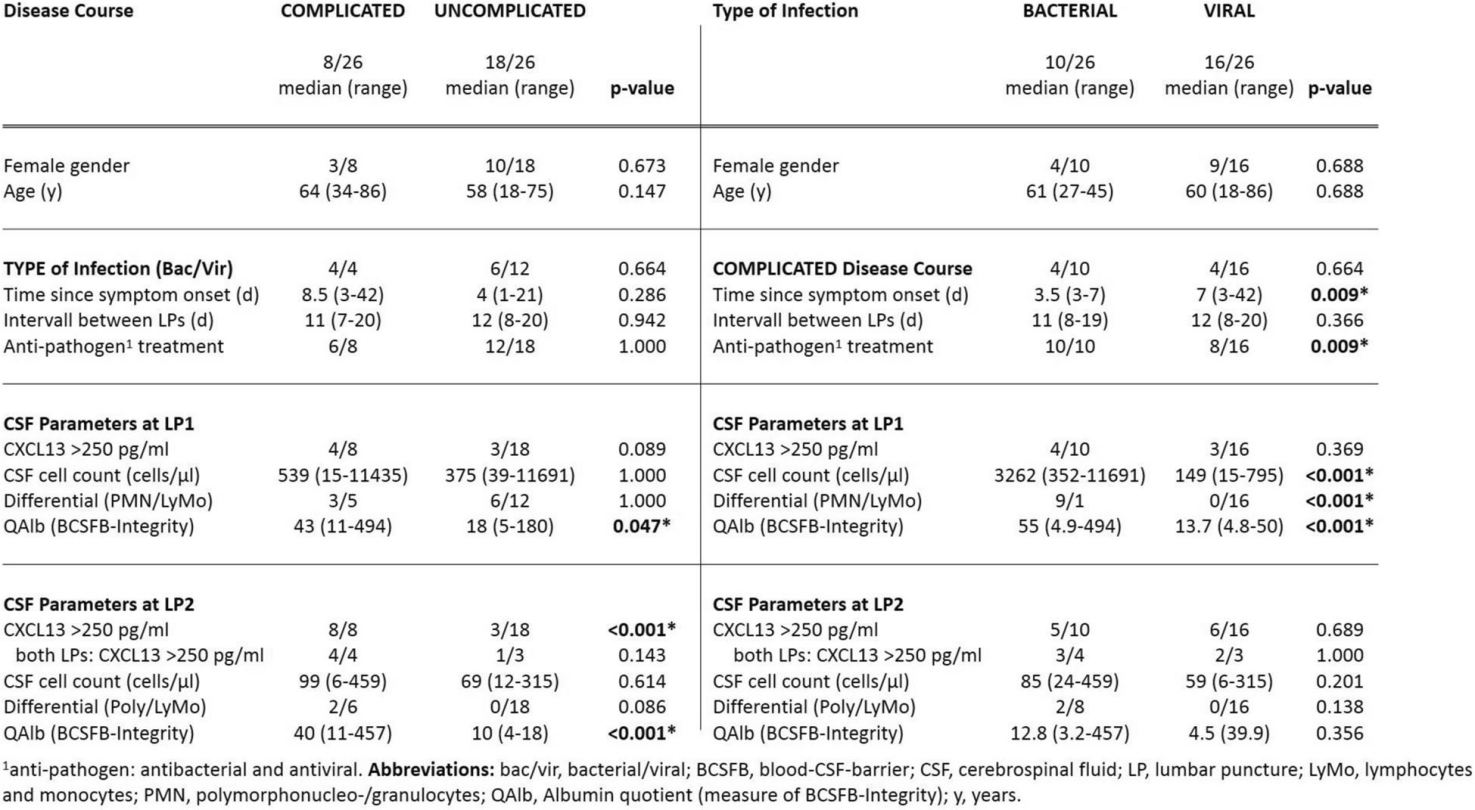
Clinical and laboratory characteristics of the study cohort stratified by disease course and type of infection

Analysis of CSF parameters measured at admission and follow-up revealed differences in CXCL13 elevations and QAlb but not CSF cell counts between groups (Table 1). In detail, half (4/8) of the patients with a complicated disease course compared to 17% (3/18) patients with an uncomplicated disease course showed CLXC13 > 250 pg/ml at the first LP. QAlb was higher in patients with a complicated disease course (median 43, range 11–494) compared to patients with an uncomplicated disease course (median 18, range 5–180; *p* = 0.047). CSF cell counts and cell differential did not differ between groups (Table 1).

At follow-up, all patients with a complicated disease course had CXCL13 elevations > 250 pg/ml (8/8; 100%), whereas the fraction of patients with an uncomplicated disease course was again 17% (3/18; *p* < 0.001). ROC analysis computed to estimate the performance of CXCL13 in discriminating between disease courses in these patients resulted in an AUC of 0.958, CI 0.888–1.000, and *p* < 0.001. The 250 pg/ml cut-off discriminated with 100% sensitivity and 83% specificity. Of note, four patients with a complicated disease course but only one of the three patients with an uncomplicated disease course had persistent CXCL13 elevations at both LPs. The other two patients with CXCL13 elevations > 250 pg/ml and uncomplicated disease course were the only patients with VZV infection not receiving aciclovir. QAlb was unaltered in patients with a complicated disease course (median 40, range 11–457) but had declined in patients with an uncomplicated disease course (median 10, range 4–18; *p* < 0.001).

CSF cell numbers had declined in both groups but again did not differ between groups nor did the cell differential (Table 1).

We next investigated the occurrence of CXCL13 elevations in patients dichotomized by type of infection to avoid bacterial or viral etiology biasing results. Ten of the 26 patients (38%) had bacterial and 16 (62%) had viral type of neuroinfections. Patient groups did not differ in regard to gender, age, disease course, and interval between LPs. Patients with viral neuroinfections, however, had a longer time since symptom onset until initial LP (*p* = 0.009) compared to patients with bacterial neuroinfections, and fewer received anti-pathogen treatment (*p* = 0.009) (Table 1).

As expected, patients with bacterial neuroinfections showed higher CSF cell counts, a predominance of granulocytes in the differential, and a higher QAlb at the initial but not at the follow-up LP. Most importantly, patient groups did not differ regarding CXCL13 elevations (Table 1).

## Discussion

With this study, we aimed at clarifying the significance of CXCL13 elevations in CSF of patients with established bacterial and viral neuroinfection other than LNB, and we report an association of CXCL13 elevations and complicated disease course.

CXCL13 elevations > 250 pg/ml occurred in all eight patients with a complicated disease course compared to every sixth patient with an uncomplicated disease course. Half of them showed pronounced CXCL13 elevations already at the initial LP persistent over time, which was the case only in a single patient with an uncomplicated disease course. The other half of patients developed CXCL13 elevations somewhere in-between the two LPs. This is in line with the fact that complications can occur early in some patients and with delay in others and suggests that repeat measurements of CXCL13 may indicate and/or confirm complications in neuroinfections.

CXCL13 elevations occurred likewise both in bacterial and viral neuroinfections, in anti-pathogen treated and untreated patients, indicating that host-intrinsic rather than pathogen-specific factors determined the course of the CNS infection. With this observation, we are in line with Fujimori et al. [12] who identified CXCL13 as prognostic markers for patients developing aseptic meningoencephalitis of unknown etiology. Such host-intrinsic factors may relate to an insufficient capability in eradicating the pathogen or controlling the inflammatory response, particularly if CXCL13 elevations develop or persist in spite of antibacterial and antiviral treatment. Whereas lack of treatment might have delayed recovery or triggered a stronger immune reaction in the two VZV patients who developed CXCL13 elevations at follow-up but had an uncomplicated disease course. The time course of CXCL13 elevations during bacterial and viral neuroinfections hence appeared different from what we learned about LNB, that elevations are pronounced and immediate and decline in response to antibiotic treatment [1, 4, 5]. In terms of the CNS immune defense, such acute CXCL13 elevations mean acute recruitment of B cells followed by a strong intrathecal humoral anti-borrelial immune response, as evidenced by laboratory diagnosis of LNB.

Rupprecht et al. identified human monocytes as possible source for CXCL13 elevations in LNB. They showed that monocytes released CXCL13 upon in vitro stimulation with lipid moieties containing a spirochetal surface motif but not with pneumococci [5]. These findings may explain the exceptional high CXCL13 elevations in LNB and other spirochetal CNS infections [1, 2, 4, 5, 7]. However, they do not disclose the source of CSF CXLC13 in other cases, as for example the pneumococci patient of our group with complicated disease course.

In bacterial and viral neuroinfections, we have little data compared to LNB and are constrained to assumptions about the underlying immune process, for example that CXCL13 elevations signal progressing infections necessitating a prolonged or intensified immune help from peripheral blood.

Bacterial and viral pathogens generally trigger different immune responses, and patients stratified by type of infection were clearly distinguishable by CSF cell counts, differential and QAlb at the initial LP but not at follow-up, when CSF cell counts had declined including a switch from predominant granulocytes to a lympho-monocyte response in bacterial infection. The interval since symptom-onset on the other hand was longer in patients with viral infection. Importantly, patient groups did not differ in frequencies of CXCL13 elevations confirming highly elevated CXCL13 associated with disease course and not type of infection.

QAlb was the only other parameter persistently and pronouncedly increased in patients with a complicated disease course discriminating well between groups. QAlb elevations alone, however, are very unspecific and mainly inform about the degree of BCSFB disruption, i.e., meningeal inflammation. CXCL13, in contrast, is a signal molecule and chemoattractant, allowing clues on the downstream immune reaction such as recruitment of B cell help in the inflamed CNS.

## Conclusion

With this observational study, we provide the first evidence that CXCL13 elevations in CSF occur during progress of infections and signal possible complications, such as parenchymal involvement seen in encephalitis, myelitis, abscesses, and vasculitis. We concede that the number of patients is small and many questions remain to be clarified. These findings, however, underline the functional role of CSF CXCL13 in the immune defense of CNS infections indicating unresolved inflammatory processes and points at an additional diagnostic value of this biomarker in alerting for complications.

## Abbreviations

AIE: Autoimmune encephalitis
bac/vir: Bacterial/viral
BCSFB: Blood-CSF barrier
CDC: Complicated disease course
CNS: Central nervous system
CSF: Cerebrospinal fluid
dysg: Dysgalacticae
KS: Kolmogorov-Smirnow
LNB: Lyme neuroborreliosis
LP: Lumbar puncture
MS: Multiple sclerosis
n.d.: Not defined
pneum: Pneumoniae
QAlb: CSF/serum albumin quotient
Staph.: *Staphylococcus*
Strept.: *Streptococcus*
TBE: Tick-borne meningoencephalitis
UDC: Uncomplicated disease course
VZV: Varicella zoster virus
